# Prevalence and factors associated with anxiety and depression after COVID-19^1^

**DOI:** 10.1590/1518-8345.8173.4915

**Published:** 2026-08-12

**Authors:** Iasmin Daniele de Araújo, Isabella Joyce Silva de Almeida Carvalho, Lusineide Carmo Andrade de Lacerda, Luciana Pessoa Maciel Diniz, Flávia Emília Cavalcante Valença Fernandes, Roxana Braga de Andrade Teles

**Affiliations:** 1 Universidade de Pernambuco, Campus Petrolina, Petrolina, PE, Brazil.; 2 Scholarship holder at the Fundação de Amparo à Ciência e Tecnologia do Estado de Pernambuco (FACEPE), Brazil.

**Keywords:** Anxiety, Depression, COVID-19, Post-Acute COVID-19 Syndrome, Mental Health, Risk Factors

## Abstract

**Objective:**

to estimate the prevalence and factors associated with anxiety and depression in patients recovering from COVID-19.

**Method:**

a cross-sectional study conducted with 381 patients four months after SARS-COV-2 infection. Sociodemographic and clinical-epidemiological data were collected, as well as information about the incidence of stressful events related to the COVID-19 pandemic, by means of a questionnaire and by applying the Hospital Anxiety and Depression Scale. Descriptive and inferential analyses with binary logistic regression were performed to identify the associated factors.

**Results:**

the prevalence of possible and probable anxiety and depression was 46.45% and 22.31%, respectively. Fear of contracting COVID-19 again, previous history of mental disorders and family history of chronic diseases were associated with higher chances of anxiety and depression. Family incomes of up to one minimum wage and presence of comorbidities were associated with depression; in turn, having a job proved to be a protective factor in the case of anxiety.

**Conclusion:**

high prevalence of anxiety and depression was observed in the post-COVID-19 period, associated with psychosocial, clinical and socioeconomic factors and evidencing the need for screening and mental health care strategies targeted at this population group.

## Highlights

**(1)** High prevalence of anxiety in patients recovered from COVID-19. **(2)** Factors like gender, economic situation, deaths and fear of COVID-19 exerted an influence on emotional health. **(3)** Social/clinical factors and pandemic-associated stress were related to anxiety and depression. **(4)** Interventions and early diagnoses are essential in post- COVID-19 psychological care.

## Introduction

Caused by SARS-CoV-2, COVID-19 was declared a pandemic in March 2020 and emerged as one of the main global health challenges of the 21^st^century^1^. Currently, there are more than 700 million confirmed cases worldwide^2^. Although the initial efforts were focused on reducing the morbidity and mortality associated with the acute phase of the disease, an increasing body of evidence indicates the persistence of physical and psychological manifestations after the infection, thus characterizing the post-COVID-19 period^3^.

The persistence of COVID-19-related symptoms can last for weeks or months. Initially, for being a new disease with limited information about its chronic manifestations, the long-term condition was paid less attention when compared to the clinical aspects inherent to the acute phase^4^. However, some of the cases proved to be clinically relevant, as patients with mild or severe forms of the disease started to present long-term symptoms and sequelae, even after at least two months of having being infected, affecting various body systems and even with psychological manifestations^5^. These prolonged COVID-19 manifestations exert negative impacts on quality of life, hindering recovery and resumption of daily activities^6^.

The physical complications in patients during the post-COVID-19 period are widely discussed; however, the long-term consequences for mental health have yet not been sufficiently focused on^5^. Recent evidence shows that the SARS-CoV-2 infection can cause alterations in the neuroimmune circuits, increasing the risk for the onset and/or worsening of comorbidities in infected individuals. In addition to that, psychosocial stress and the severe form of the disease are risk factors for developing signs and symptoms indicative of psychological disorders^7^.

COVID-19 infection is associated with mental overload as a result of stressful events, insecurity, anxiety and fear, contributing to the development of psychological disorders^8^. These disorders can be understood as manifestations of the stressful events experienced by COVID-19 victims, triggering anxiety and depression signs and symptoms in approximately 30% to 40% of the patients during the post-infection period^9^. A cross-sectional study with 70 participants evidenced that only eight (11.4%) did not present symptoms related to depression, which varied from mild to moderate. In relation to anxiety, it was observed that 18 individuals (25.7%) presented symptoms associated with the disease after COVID-19^7^.

Considering the high prevalence of anxiety and depression in the post-COVID-19 period^10^, as well as their physical, mental and social repercussions that contribute to distancing from work, to productivity losses and to reduced quality of life, in addition to the scarcity of studies found in the literature, research studies on mental health state changes among COVID-19 survivors become necessary. In this context, the objective of the current study is to estimate the prevalence and factors associated with anxiety and depression in patients recovering from COVID-19.

## Method

### Study design

This is an analytical study of the cross-sectional type based on the Strengthening the Reporting of Observational Studies in Epidemiology (STROBE) guidelines^11^.

### *Study* locus *and period*

The study was conducted between September 2022 and July 2023 in a municipality from Petrolina (Pernambuco), at the University Hospital belonging to *Universidade Federal do Vale do São Francisco*(*Hospital Universitário da Universidade Federal do Vale do São Francisco*, HU-UNIVASF) and in the COVID-19 Screening Center.

### Population

The study population was comprised by patients with a history of COVID-19 infection, 4 months after having the disease diagnosed.

### Selection criteria

The individuals included in the sample were aged at least 18 years old, of both genders and had their COVID-19 diagnoses confirmed by means of the Polymerase Chain Reaction (PCR) test (or RT-PCR) for the SARS-CoV-2 virus, with a minimum of four months since the infection. People with cognitive deficits that prevented them from understanding the research instruments were excluded, as well as those who had not answered the instruments in full, thus precluding data processing and analysis.

### Participants

Sample calculation was performed based on the estimated number of people affected by COVID-19 in 2022 in the municipality of Petrolina, considering a 95% confidence level. The sample size was calculated using the OpenEpi tool (http://www.OpenEpi.com), resulting in a sample comprised by 381 individuals.

### Study variables

The primary study outcome was prevalence of anxiety and depression. The independent variables included sociodemographic and clinical-epidemiological characteristics, as well as stressful events related to the COVID-19 pandemic.

### Instruments used to collect the information

A structured questionnaire prepared by the researchers was used to collect the sociodemographic and clinical-epidemiological information, as well as the incidence of stressful events related to the COVID-19 pandemic, such as unemployment, grief and changes in family structure. In addition, the Hospital Anxiety and Depression Scale (HADS) was also used. It was possible to self-apply both instruments, under due supervision by the researcher. In the case of participants with reading difficulties, the interviewer read the questions in full, along with the respective answer options.

HADS is an adapted instrument that has been validated to be used in Brazil and presents good parameters to screen anxiety and depression symptoms^12^. Initially developed to assess these symptoms in patients admitted to non-psychiatric clinical hospitals, the scale was later on started to also be used in non-hospitalized individuals without comorbidities^13^.

The scale has 14 items distributed into two subscales: Anxiety (HADS-A) and Depression (HADS-D), with seven items each. Each item has four answer options, with scores varying from zero to three and totaling 21 points for each subscale. The final score is calculated by adding up the values indicated by the participants. The scores can also be categorized according to the degree to which the symptoms are manifested (normal, mild, moderate and severe). In this study, cases with scores above 8, 11 and 15 points are considered as possible, probable and severe, respectively^14^.

### Data collection

For data collection, an analysis of the HU-UNIVASF patients’ medical records and of the COVID-19 Screening Center data (made available by the Municipal Health Department) was initially performed to select the individuals that met the study eligibility criteria. The subjects selected were then invited to take part in the research and, after they accepted, the researcher conducted individual semi-structured interviews. These interviews took place in the participants’ homes or in a private environment and lasted a mean of 20 minutes.

### Data analysis

The variables were tabulated in Microsoft Office Excel^®^ 2016 for data recording and coding. Subsequently, all the information was exported to the Stata statistical program (version 16.0) to perform the descriptive analysis and for the association between the variables. In the descriptive analysis, absolute and relative frequency calculations were used for the categorical variables; in turn, central tendency and dispersion measures were calculated for the numerical variables.

The inferential analysis of the factors associated with anxiety and depression was performed by means of binary logistic regression. For this stage, the outcome variables were dichotomized into presence of anxiety and depression (possible or probable cases: scores ≥8 points versus unlikely cases), according to the classification obtained in HADS. As a first step, bivariate analyses were performed between each outcome and the sociodemographic/clinical-epidemiological variables and those related to stressful events, using Pearson’s chi-square or Fisher’s exact test and adopting p-values<0.20 as criterion to select the variables that were likely to be included in the multiple models.

Subsequently, binary logistic regression modeling with “stepwise forward” automatic selection was implemented, including in the initial model all variables with p<0.20 in the bivariate analysis. The variables that remained in the model were those associated with the outcome at a 5% significance level, with adjusted Odds Ratio (OR) estimates and their respective 95% confidence intervals. Fit and performance of the models were assessed using the Hosmer-Lemeshow test.

### Ethical aspects

The research was approved by the Research Ethics Committee of the Amaury de Medeiros Integrated Health Center (*Comitê de* Ética *em Pesquisa do Centro Integrado de Saúde Amaury de Medeiros*, CEP-CISAM/UPE), under Opinion No. 5,760,468 and registered in Certificate of Submission for Ethical Appraisal (CAAE) No. 57873222.1.0000.5191.

## Results

The study participants were 381 individuals with a mean age of 36.72 years old (±13.14) and mean post-COVID-19 infection time of 15.19 months (±8.73). There was predominance of the female gender (65.62%), of people self-declared as brown-skinned (62.29%), single (42.74%) and with Complete High School (40.16%). In relation to their economic situation, 75.99% of the participants were working, with family incomes above 2 minimum wages in 42.33% of the cases (Table 1).

Most of the subjects reported family history of chronic diseases (53.16%) and had no personal history of mental disorders (87%), of respiratory diseases (70.53%) or of other associated comorbidities (83.11%). As for COVID-19 treatment, 98.15% of the patients underwent it at their homes, as they were classified as mild cases (Table 1).

Regarding the pandemic-related stressful events, it was observed that most of the participants (79.26%) had not experienced losses related to COVID-19 or to other diseases (70.47%). When asked about fearing COVID-19 reinfection, 50.66% assented. In addition to that, most of the participants worked during the pandemic (71.43%) and 57.56% contributed to the family income (Table 1).

**Table 1:** Sociodemographic/Clinical-epidemiological data and stressful events related to the pandemic among patients affected by COVID-19. Petrolina, PE, Brazil, 2023

Variables	n*	%^†^
Anxiety		
Unlikely	204	53.54
Possible	99	25.98
Probable	78	20.47
Depression		
Unlikely	296	77.69
Possible	61	16.01
Probable	24	6.30

*SD = Standard Deviation; ^†^n = Absolute number; ^‡^% = Relative frequency; ^§^Minimum wage in force = BR$ 1,320.00, Brazil, 2023

When analyzing the scores obtained in the Hospital Anxiety and Depression Scale (HADS), it was verified that 53.54% (n=204), 25.98% (n=99) and 20.47% (n=78) of the participants presented the Unlikely, Possible and Probable anxiety profiles, respectively. As for depression, the results showed that 77.69% (n=296), 16.01% (n=61) and 6.30% (n=24) were unlikely, possible and probable cases, respectively (Table 2). For the purposes of the inferential statistical analysis, the “Possible” and “Probable” categories were grouped, thus constituting a dichotomous variable (Unlikely versus Possible/Probable). This strategy was adopted to meet the statistical assumptions and improve robustness of the analyses. Thus, the prevalence of Possible/Probable anxiety was 46.45%, whereas it was 22.31% for Possible/Probable depression.

A statistically significant association was observed between anxiety and the following variables: gender, economic situation, family income, family history, presence of respiratory diseases, history of mental disorders, fear of contracting COVID-19 again and receiving government aids (p<0.05) (Table 3).

As for depression, a statistically significant difference was verified with these variables: gender, family income, family history, presence of comorbidities, history of mental disorders, death of family member(s) due to COVID-19, fear of contracting COVID-19 again and receiving government aids (p<0.05) (Table 3).

**Table 2:** Analysis of the Hospital Anxiety and Depression Scale among patients affected by COVID-19. Petrolina, PE, Brazil, 2023

Variables	n*	%^†^
Anxiety		
Unlikely	204	53.54
Possible	99	25.98
Probable	78	20.47
**Depression**		
Unlikely	296	77.69
Possible	61	16.01
Probable	24	6.30

*n = Absolute number; ^†^% = Relative frequency

**Table 3:** Association of anxiety and depression with sociodemographic/clinical-epidemiological characteristics and stressful events related to the pandemic among patients affected by COVID-19. Petrolina, PE, Brazil, 2023

Variables	Anxiety				p-value	Depression				p-value
	Unlikely		Possible/Probable			Unlikely		Possible/Probable		
	n*	%^†^	n*	%^†^		n*	%^†^	n*	%^†^	
**Gender**										
Male	94	46.1	37	20.9	<0.001^‡^	116	39.2	15	17.7	<0.001^‡^
Female	110	53.9	140	79.1		180	60.8	70	82.4
**Marital status (married/stable union)**										
Not married/stable union	101	49.5	95	53.7	0.417^‡^	148	50.0	48	56.5	0.293^‡^
Married/Stable union	103	50.5	82	46.3		148	50.0	37	43.5
**Schooling level (low)**										
≥ High School	177	86.8	158	89.3	0.455^‡^	260	87.8	75	88.2	0.921^§^
≤ Elementary School	27	13.2	19	10.7		36	12.2	10	11.8
**Ethnicity**										
White	55	27.0	39	22.0	0.266^‡^	76	25.7	18	21.2	0.396^‡^
Not white	149	73.0	138	78.0		220	74.3	67	78.8
**Economic situation**										
Unemployed	36	17.7	57	32.2	0.001^‡^	71	24.0	22	25.9	0.720^‡^
Employed	168	82.4	120	67.8		225	76.0	63	74.1
**Family income (up to 1 MW)^||^**										
>1 minimum wage	169	82.8	129	72.9	0.019^‡^	241	81.4	57	67.1	0.005^‡^
≤1 minimum wage	35	17.2	48	27.1		55	18.6	28	32.9
**Family history**										
No	110	53.9	68	38.6	0.003^‡^	149	50.5	29	34.1	0.008^‡^
Yes	94	46.1	108	61.4		146	49.5	56	65.9
**Comorbidities**										
No	174	85.3	141	80.6	0.221^‡^	252	85.1	63	75.9	0.047^‡^
Yes	30	14.7	34	19.4		44	14.9	20	24.1
**Respiratory diseases**										
No	154	75.5	114	64.8	0.022^‡^	213	72.2	55	64.7	0.182^‡^
Yes	50	24.5	62	35.2		82	27.8	30	35.3
**Hospitalization**										
No	199	98.0	172	98.3	0.854^§^	287	97.6	84	100.0	0.356^§^
Yes	4	2.0	3	1.7		7	2.4	0	0.0
**History of mental disorders**										
No	192	95.1	136	77.7	<0.001^‡^	265	90.4	63	75.0	<0.001^‡^
Yes	10	5.0	39	22.3		28	9.6	21	25.0
**Death of family member(s) due to COVID-19**										
No	166	81.8	136	76.8	0.235^‡^	242	81.8	60	71.4	0.039^‡^
Yes	37	18.2	41	23.2		54	18.2	24	28.6
**Death of family member(s) due to other diseases**										
No	134	66.7	125	71.4	0.320^‡^	205	70.2	54	64.3	0.302^‡^
Yes	67	33.3	50	28.6		87	29.8	30	35.7
**Fear of contracting COVID19 again**										
No	130	64.4	62	35.0	<0.001^‡^	168	57.1	24	28.2	<0.001^‡^
Yes	72	35.6	115	65.0		126	42.9	61	71.8
**Worked during the pandemic**										
No	55	27.2	53	30.1	0.536^‡^	82	28.0	26	30.6	0.640^‡^
Yes	147	72.8	123	69.9		211	72.0	59	69.4
**Government aids**										
No	184	92.0	140	81.9	0.003^‡^	260	89.7	64	79.0	0.011^‡^
Yes	16	8.0	31	18.1		30	10.3	17	21.0
**Is the breadwinner**										
No	149	73.0	125	70.6	0.600^‡^	218	73.7	56	65.9	0.160^‡^
Yes	55	27.0	52	29.4		78	26.4	29	34.1

*n = Absolute number; ^†^% = Relative frequency; ^‡^Chi-square; ^§^Fisher’s Exact; ^||^Minimum wage in force = BR$ 1,320.00, Brazil, 2023

The anxiety-associated factors evidenced in the regression models indicated that fear of contracting COVID-19 again was associated with higher chances of anxiety (Possible/Probable) (OR=3.08; 95%CI: 1.95-4.88; p<0.001), as well as with previous history of mental disorders (OR=5.56; 95%CI: 2.42-12.76; p<0.001). The participants’ economic situation presented an inverse association, with lower chances of anxiety among the participants that had a job and characterizing a protective effect, with an approximate 46% reduction in the anxiety chances in relation to the other categories (OR=0.54; 95%CI: 0.31-0.93; p=0.027). Increased chances of anxiety were also observed in the presence of family history of chronic diseases (OR=1.76; 95%CI: 1.11-2.79; p=0.016) (Table 4).

As for depression, an association was detected with fear of contracting COVID-19 (Possible/Probable), with OR=3.24 (95%CI: 1.85-5.69; p<0.001). In addition to that, the participants that presented higher depression chances were those with a history of mental disorders (OR=2.80; 95%CI: 1.40-5.58; p=0.003), family incomes of up to one minimum wage (OR=2.18; 95%CI: 1.21-3.93; p=0.009), family history of chronic diseases (OR=1.81; 95%CI: 1.04-3.13; p=0.035) and presence of comorbidities (OR=1.97; 95%CI: 1.03-3.78; p=0.041), as presented in Table 4.

**Table 4:** Binary logistic regression models corresponding to the factors associated with anxiety and depression among patients affected by COVID-19. Petrolina, PE, Brazil, 2023

Variables	Anxiety				Depression			
	Adjusted OR*	95%CI^†^		p-value	Adjusted OR*	95%CI^†^		p-value
Fear of contracting COVID-19 again								
Yes vs.^‡^ No	3.08	1.95	4.88	<0.001	3.24	1.85	5.69	<0.001
History of mental disorders								
Yes vs.^‡^ no	5.56	2.42	12.76	<0.001	2.80	1.40	5.58	0.003
Economic situation								
Employed	0.54	0.31	0.93	0.027				
Family history of chronic diseases								
Yes vs.^‡^ no	1.76	1.11	2.79	0.016	1.81	1.04	3.13	0.035
Family income^§^ up to 1 minimum wage								
≤1 minimum wage vs.^‡^ >1 minimum wage	1.69	0.96	2.97	0.071	2.18	1.21	3.93	0.009
Time since COVID-19 diagnosis (months)								
1-month increase	0.98	0.95	1.01	0.133				
Comorbidities								
Yes vs.^‡^ no					1.97	1.03	3.78	0.041

*OR = Odds Ratio; ^†^95%CI = 95% Confidence Interval; ^‡^vs. - Versus; Hosmer-Lemeshow test for fit of the model Anxiety: chi²(8)=2.12 and p=0.9773; Depression: chi²(8)=13.92 and p=0,0839; ^§^Minimum wage in force = BR$ 1,320.00, Brazil, 2023

## Discussion

This study identified high prevalence of anxiety and depression symptoms among individuals evaluated after the SARS-CoV-2 infection, indicating that mental health changes persist during the post-acute COVID-19 period. These outcomes were associated with sociodemographic, clinical and psychosocial characteristics, with female gender, socioeconomic vulnerability and persistent fear of reinfection standing out.

The prevalence of anxiety found was higher than the global estimated 25% increase in the first year of the COVID-19 pandemic^3^, as well as than those described for the Brazilian population in general before and during the pandemic, which point to an approximate 14.3% prevalence for anxiety and to values between 4% and 5% in the case of depression^15^ ^-^ ^16^. This finding constitutes a relevant aspect of the study, as it evidences that individuals in the post-COVID-19 period remained at an increased risk of psychological distress in relation to the general population. When compared to a Brazilian cohort of hospitalized patients using the same instrument, the prevalence values found were different^10^, which can be related to contextual and methodological differences. The current sample was predominantly comprised by mild cases followed-up in the home context, which may have favored the persistence of less clinically intense psychological symptoms, though long-lasting.

The results proved to be coherent with diverse international longitudinal evidence. Studies conducted with COVID-19 survivors followed-up after hospital treatments identified higher prevalence values for anxiety (42%) and depression (31%)^17^, suggesting that baseline clinical severity can modulate these outcomes. On the other hand, cohort studies with prolonged follow-up periods showed that, although the depressive symptoms presented a decreasing trend throughout time, the anxiety levels remained unchanged, indicating that the latter is more persistent than depression during the post-COVID-19 period^18^. In this context, recent evidence indicated that anxiety and depression tend to manifest themselves concomitantly and that they act like predictive factors of the post-COVID-19 condition, reinforcing the hypothesis that psychological distress is part of the clinical spectrum of long COVID^5^.

In addition to the high prevalence values, the logistic regression models evidenced that fear of contracting COVID-19 again and previous history of mental disorders were the main factors associated both with anxiety and with depression, increasing the chances of these outcomes by approximately three times. In addition, family incomes of up to one minimum wage, family history of chronic diseases and presence of comorbidities were associated with higher depression chances; in turn, having a job proved to be a protective factor against anxiety.

Higher prevalence of anxiety and depression was noticed among women, a result that is similar to the one described in national^10^ ^,^ ^17^ and international^19^ studies. This higher vulnerability to suffering mental disorders in the female gender when compared to men was also identified in the Brazilian population during the COVID-19 pandemic^17^. This finding is in consonance with global reports that point to risk levels between 1.5 and 2 times higher for mental disorders among women^3^. In addition, a recent meta-analysis involving COVID-19 survivors identified higher anxiety (OR=2.1) and depression (OR=1.8) chances among women, reinforcing that gender inequalities persisted in the post-pandemic context^19^. This higher vulnerability among women was attributed to overload of household and care activities, to economic insecurity and to intensified psychosocial impacts during the pandemic^19^.

As for the socioeconomic determinants, the findings evidenced that having a job exerted a protective effect against anxiety, whereas low family incomes significantly increased the depression chances. These results were in line with recent longitudinal studies which showed that unemployment increased up to 2.4 times the anxiety chances and that low incomes doubled the depression risk in prolonged follow-ups after COVID-19^20^. Similarly, a study about post-pandemic evolutions identified that family incomes ≤1 minimum wage increased 2.1 times the depression chances, whereas having a job maintained its protective effect at around 40%, corroborating the pattern observed in this research study^21^.

Previous history of mental disorders stood out as one of the factors presenting the strongest associations with anxiety and depression; this result in consistent with longitudinal three-year analyses that indicated high recurrence or persistence of mental disorders after COVID-19 in individuals with previous psychiatric vulnerability^22^. Additional evidence indicated that people with previous mental disorders presented up to five times higher risks of developing anxiety and depression symptoms in the long COVID context^23^.

Similarly, the association between chronic diseases, comorbidities and depressive symptoms observed in this study proved to be coherent with a number of meta-analysis that indicated higher anxiety and depression chances among individuals with chronic conditions, with ORs varying from 1.7 to 2.0, possibly mediated by persistent neuroinflammation mechanisms^5^. Some studies also showed that presence of comorbidities almost doubled the depression chances (OR=1.9) and that respiratory diseases increased the anxiety risk (OR=1.6), reinforcing the interaction between physical ailments and psychological distress during the post-COVID-19 period^24^.

The multiple models showed that patients with a family history of comorbidities and a personal history of mental disorders presented higher anxiety and depression chances, in consonance with epidemiological studies which point to psychiatric antecedents as an important vulnerability factor for the development or intensification of psychological disorders after the infection^5^ ^,^ ^22^ ^-^ ^23^. In addition, presence of clinical comorbidities almost quadrupled the depression chances, reinforcing the role of chronic ailments in these patients’ emotional overload.

In addition to that, the fear of contracting SARS-CoV-2 again was strongly associated both with anxiety and with depression in this study. A research study conducted in the state of Mato Grosso do Sul verified that 64% of the participants reported fear of contracting the disease again^24^, a condition that tends to increase emotional and physical burden, thus favoring psychological distress. International studies evidenced that fear of contracting COVID-19 again emerged as the main predictor of anxiety and depression; this finding is consistent with surveys on mental health evolutions that identified a 2.8 to 3.5-fold increase in the chances for both outcomes associated with persistent fear^21^. A specific meta-analysis about psychological factors in long COVID reinforced this result by showing that the reinfection fear increased approximately three times the anxiety chances, emerging as one of the most robust determinants of prolonged psychological distress^5^.

The main limitations of the current study were as follows: its cross-sectional design, which precluded causal inferences; and the predominance of mild cases, which limits generalization of the results to populations presenting higher clinical severity levels. In addition to that, the scarcity of national studies with a similar design restricted broader comparisons.

Despite these limitations, this study contributed to advancing scientific knowledge in the Health and Nursing areas because it produced national evidence on the prevalence and factors associated with anxiety and depression during the post-COVID-19 period, in a population predominantly comprised by mild cases followed-up in an out-of-hospital context. As they integrate sociodemographic, clinical and psychosocial variables in adjusted analytical models, the findings expanded what is known about the determinants of psychological distress after the SARS-CoV-2 infection. This body of evidence strengthens the scientific grounds to develop screening, follow-up and comprehensive mental health care strategies, with direct repercussions for the care practice, planning in health and Nursing knowledge production.

## Conclusion

The study identified high prevalence of anxiety among COVID-19 survivors, with more chances among people fearing new contagions, personal history of mental disorders, family history of chronic diseases, low incomes and unemployed, whereas having a job showed a protective effect. Depression presented a lower prevalence value than the one found for anxiety, but it was mainly associated with reinfection fear, history of mental disorders, low incomes, family history of chronic diseases and presence of clinical comorbidities.

These findings reinforce the need for strategies targeted at systematically screening anxiety and depression symptoms in the population after COVID-19, with a priority focus on socially vulnerable groups and on individuals with psychiatric antecedents. They also underscore the importance of offering psychosocial interventions that may reduce the impact exerted by COVID-19 on mental health.

## Data Availability

All data generated or analysed during this study are included in this published article.
