# Supplementary material for: Prevalence and factors associated with anxiety and depression after COVID-19
Source: Rev Lat Am Enfermagem. 2026 Aug 12;34:e4915. doi: 10.1590/1518-8345.8173.4915 (PMC13505607; doi:10.1590/1518-8345.8173.4915)
Supplement: Translation

# Translation: Prevalência e fatores associados à ansiedade e à depressão pós-COVID-19^*^

**DOI:** 10.1590/1518-8345.8173.4916

**Authors:** Iasmin Daniele de Araújo, Isabella Joyce Silva de Almeida Carvalho, Lusineide Carmo Andrade de Lacerda, Luciana Pessoa Maciel Diniz, Flávia Emília Cavalcante Valença Fernandes, Roxana Braga de Andrade Teles

**Affiliations:** 1 Universidade de Pernambuco, Campus Petrolina, Petrolina, PE, Brasil.; 2 Bolsista da Fundação de Amparo à Ciência e Tecnologia do Estado de Pernambuco (FACEPE), Brasil.

**Keywords:** Ansiedade, Depressão, COVID-19, Síndrome Pós-COVID-19 Aguda, Saúde Mental, Fatores de Risco.

## Abstract

**Objetivo:**

estimar a prevalência e os fatores associados à ansiedade e à depressão em pacientes que se recuperam da COVID-19.

**Método:**

estudo transversal, realizado com 381 pacientes, após quatro meses da infecção pelo SARS-COV-2. Foram coletados dados sociodemográficos e clínico-epidemiológicos, bem como informações sobre a ocorrência de eventos estressantes relacionados à pandemia de COVID-19, por meio de questionário e aplicação da Escala Hospitalar de Ansiedade e Depressão. Realizaram-se análises descritivas e inferenciais, com regressão logística binária para a identificação dos fatores associados.

**Resultados:**

a prevalência de ansiedade possível e provável foi de 46,45%, e a de depressão possível e provável, de 22,31%. O medo de contrair COVID-19 novamente, histórico prévio de transtorno mental e antecedentes familiares de doenças crônicas estiveram associados a maiores chances de ansiedade e depressão. Renda familiar de até um salário mínimo e presença de comorbidades associaram-se à depressão, enquanto estar empregado mostrou-se fator protetor para a ansiedade.

**Conclusão:**

observou-se elevada prevalência de ansiedade e depressão no período pós-COVID-19, associada a fatores psicossociais, clínicos e socioeconômicos, evidenciando a necessidade de estratégias de rastreamento e cuidado da saúde mental nessa população.

## Destaques

**(1)** Alta prevalência de ansiedade em pacientes recuperados da COVID-19. **(2)** Fatores como sexo, situação econômica, perdas e medo da COVID-19 influenciam a saúde emocional. **(3)** Fatores sociais, clínicos e o estresse associado à pandemia estão relacionados à ansiedade e à depressão. **(4)** Intervenções e diagnóstico precoce são essenciais no cuidado psicológico pós-COVID-19.

## Introdução

A COVID-19, causada pelo SARS-CoV-2, foi declarada pandemia em março de 2020, configurando-se como um dos principais desafios sanitários globais do século XXI^1^. Atualmente, contabilizam-se mais de 700 milhões de casos confirmados em nível global^2^. Embora os esforços iniciais tenham se concentrado na redução da morbimortalidade associada à fase aguda da doença, evidências crescentes indicam a persistência de manifestações físicas e psicológicas após a infecção, caracterizando o período pós-COVID-19^3^. 

A persistência de sintomas relacionados à COVID-19 pode perdurar por semanas ou meses. Inicialmente, por ser uma doença nova e com informações limitadas sobre suas manifestações crônicas, o quadro a longo prazo recebeu menor atenção quando comparado à clínica da fase aguda^4^. Contudo, parte dos casos mostrou-se clinicamente relevante, uma vez que pacientes com quadros leves ou graves passaram a apresentar sintomas e sequelas a longo prazo, mesmo após pelo menos dois meses da infecção, acometendo diversos sistemas orgânicos, inclusive com manifestações psicológicas^5^. Essas manifestações prolongadas da COVID-19 impactam negativamente a qualidade de vida, dificultando a recuperação e o retorno às atividades diárias^6^.

As complicações físicas em pacientes no período pós-COVID-19 são amplamente discutidas; entretanto, as consequências para a saúde mental a longo prazo ainda não receberam o destaque necessário^5^. Evidências recentes mostram que a infecção pelo SARS-CoV-2 pode causar alterações nos circuitos neuro imunes, aumentando o risco de aparecimento e/ou agravamento de comorbidades em indivíduos infectados. Além disso, o estresse psicossocial e a forma grave da doença são fatores de risco para o desenvolvimento de sinais e sintomas de distúrbios psicológicos^7^. 

A infecção por COVID-19 está associada a uma sobrecarga mental decorrente de eventos estressores, insegurança, ansiedade e medo, contribuindo para o desenvolvimento de distúrbios psicológicos^8^. Esses distúrbios podem ser compreendidos como manifestações dos eventos estressantes vivenciados pelas vítimas da COVID-19, ocasionando sintomas e sinais de ansiedade e depressão em aproximadamente 30% a 40% dos pacientes no período pós-infecção^9^. Um estudo transversal com 70 participantes evidenciou que apenas oito (11,4%) não apresentavam sintomas relacionados à depressão, que variou de leve a moderada. Em relação à ansiedade, observou-se que, após a COVID-19, 18 indivíduos (25,7%) apresentaram sintomas de ansiedade associados à doença^7^. 

Tendo em vista a alta prevalência de ansiedade e depressão no período pós-COVID-19^10^, bem como suas repercussões físicas, mentais e sociais, que contribuem para o afastamento laboral, a perda da produtividade e a redução da qualidade de vida, além da escassez de estudos existentes na literatura, fazem-se necessárias investigações sobre as mudanças no estado mental entre sobreviventes da COVID-19. Nesse contexto, o presente estudo tem como objetivo estimar a prevalência e os fatores associados à ansiedade e à depressão em pacientes que se recuperam da COVID-19.

## Método

### Delineamento do estudo

Trata-se de um estudo analítico, do tipo transversal, baseado nas diretrizes do *Strengthening the Reporting of Observational Studies in Epidemiology* (STROBE)^11^.

### Local de estudo e período

O estudo foi realizado no município de Petrolina, Pernambuco, no Hospital Universitário da Universidade Federal do Vale do São Francisco (HU-UNIVASF) e no Centro de Triagem da COVID-19, no período de setembro de 2022 a julho de 2023.

### População

A população do estudo foi composta por pacientes com histórico de infecção por COVID-19, após 4 meses do diagnóstico da doença.

### Critérios de seleção

Foram incluídos na amostra indivíduos com idade igual ou superior a 18 anos, de ambos os sexos, com diagnóstico confirmado de COVID-19 por meio de teste de Reação em Cadeia da Polimerase (PCR) ou RT-PCR para o vírus SARS-CoV-2, com no mínimo quatro meses após a infecção. Foram excluídos indivíduos com déficit cognitivo que impossibilitasse a compreensão dos instrumentos de pesquisa, bem como aqueles com instrumentos preenchidos de forma incompleta, inviabilizando o processamento e a análise de dados. 

### Participantes

O cálculo amostral foi realizado com base na estimativa de pessoas acometidas pela COVID-19 no ano de 2022 do município de Petrolina, considerando um nível de confiança de 95%. O tamanho da amostra foi calculado por meio da ferramenta *OpenEpi* (http://www.OpenEpi.com), resultando em uma amostra de 381 indivíduos.

### Variáveis do estudo

O desfecho primário do estudo foi a prevalência de ansiedade e depressão. As variáveis independentes incluíram características sociodemográficas, características clínico-epidemiológicas, bem como eventos estressantes relacionados à pandemia da COVID-19.

### Instrumentos utilizados para a coleta de informações

Utilizou-se um questionário estruturado elaborado pelos pesquisadores para coletar informações sociodemográficas, clínico-epidemiológicas e a ocorrência de eventos estressantes relacionados à pandemia de COVID-19, tais como desemprego, luto e mudanças na composição familiar. Além disso, foi utilizada a Escala Hospitalar de Ansiedade e Depressão (*Hospital Anxiety and Depression Scale* - HADS). Ambos puderam ser auto aplicados, sob supervisão da pesquisadora. Para participantes com dificuldade de leitura, as perguntas foram lidas integralmente pela entrevistadora, juntamente com as respectivas opções de resposta. 

A HADS é um instrumento adaptado e validado para uso no Brasil, apresentando bons parâmetros para rastrear sintomas de ansiedade e depressão^12^. Inicialmente desenvolvida para avaliar esses sintomas em pacientes de hospitais clínicos não-psiquiátricos, a escala passou posteriormente a ser utilizada também em indivíduos não internados e sem comorbidades^13^. 

A escala é composta por 14 itens, distribuídos em duas subescalas: ansiedade (HADS-A) e depressão (HADS-D), cada uma contendo sete itens. Cada item possui quatro alternativas de resposta, com pontuação variando de zero a três, totalizando 21 pontos para cada subescala. O escore final é calculado somando-se os valores atribuídos pelos participantes. Ademais, os escores podem ser categorizados de acordo com o grau em que a sintomatologia está presente (normal, leve, moderado e grave). Neste estudo, consideraram-se como casos possíveis aqueles com pontuação superior a 8 pontos, casos prováveis aqueles com pontuação superior a 11 pontos, e casos graves aqueles com pontuação acima de 15 pontos^14^.

### Coleta de dados

Para a coleta de dados foi realizada, primeiramente, análise de prontuários dos pacientes do HU-UNIVASF e dos dados do Centro de Triagem da COVID-19, disponibilizados pela Secretaria Municipal de Saúde, para a seleção dos indivíduos que atendiam aos critérios de elegibilidade do estudo. Os selecionados foram então convidados a participar da pesquisa, e, mediante aceite, foi realizada entrevista semiestruturada individual, conduzida pela pesquisadora. As entrevistas ocorreram nas residências dos participantes ou em ambiente reservado, com duração média de 20 minutos.

### Análise dos dados

As variáveis foram tabuladas no *Microsoft Office Excel* ^*®*^ 2016 para registro e codificação dos dados. Posteriormente, as informações foram transportadas para o programa estatístico *Stata*, versão 16.0, para a realização da análise descritiva e de associação entre as variáveis. Na análise descritiva, foi utilizado o cálculo da frequência absoluta e relativa para as variáveis categóricas, e para as numéricas foram realizadas medidas de tendência central e dispersão.

A análise inferencial dos fatores associados à ansiedade e à depressão foi realizada por meio de regressão logística binária. Para essa etapa, as variáveis de desfecho foram dicotomizadas em presença de ansiedade e presença de depressão (casos possíveis ou prováveis: pontuação ≥ 8 pontos *versus* casos improváveis), conforme classificação obtida pela HADS. Inicialmente, foram conduzidas análises bivariadas entre cada desfecho e as variáveis sociodemográficas, clínico-epidemiológicas e relacionadas a eventos estressantes, utilizando-se os testes qui-quadrado de Pearson ou exato de Fisher, adotando-se o valor de p < 0,20 como critério para seleção das variáveis candidatas à inclusão nos modelos múltiplos. 

Em seguida, procedeu-se à modelagem por regressão logística binária com seleção automática do tipo *stepwise forward*, incluindo-se no modelo inicial todas as variáveis com p < 0,20 na análise bivariada. Permaneceram no modelo final aquelas associadas ao desfecho em nível de significância de 5%, com estimativa das razões de chances ajustadas (*Odds Ratio* - OR) e respectivos intervalos de confiança de 95%. O ajuste e o desempenho dos modelos foram avaliados por meio do teste de Hosmer-Lemeshow. 

### Aspectos éticos

A pesquisa foi aprovada pelo Comitê de Ética em Pesquisa do Centro Integrado de Saúde Amaury de Medeiros (CEP-CISAM/UPE), sob o parecer n°5.760.468, registrado no Certificado de Apresentação para Apreciação Ética (CAAE) n°57873222.1.0000.5191.

## Resultados

Participaram do estudo 381 indivíduos, com média de idade de 36,72 anos (±13,14) e tempo médio pós-infecção por COVID-19 de 15,19 meses (± 8,73). Observou-se predominância do sexo feminino (65,62%), indivíduos autodeclarados pardos (62,29%), solteiros (42,74%) e com ensino médio completo (40,16%). Em relação à situação econômica, 75,99% dos participantes estavam trabalhando, com renda familiar superior a 2 salários mínimos em 42,33% dos casos (Tabela 1). 

A maioria relatou antecedentes familiares de doenças crônicas (53,16%), não apresentava histórico pessoal de transtorno mental (87%), nem de doenças respiratórias (70,53%) ou outras comorbidades associadas (83,11%). Quanto ao tratamento da COVID-19, 98,15% dos pacientes foram tratados em casa, sendo classificados como casos leves (Tabela 1). 

No que se refere aos eventos estressantes relacionados à pandemia, observou-se que a maioria dos participantes (79,26%) não vivenciou perdas relacionadas à COVID-19 nem perdas por outras doenças (70,47%). Quando questionados sobre o medo de uma nova infecção por COVID-19, 50,66% declararam sentir medo. Além disso, a maioria trabalhou durante a pandemia (71,43%), e 57,56% contribuíam com o sustento da família (Tabela 1).

**Tabela 1:** Dados sociodemográficos, clínico-epidemiológicos e eventos estressantes relacionados à pandemia entre pacientes acometidos pela COVID-19. Petrolina, PE, Brasil, 2023

Variáveis	Média	DP*
Idade (anos)	36,72	13,14
Tempo COVID-19 (meses)	15,19	8,73
**Sociodemográficos**			
Variáveis	n^†^	%^‡^
Sexo		
Masculino	131	34,38
Feminino	250	65,62
Estado civil		
Solteiro	162	42,74
União Estável	41	10,82
Casado	144	37,99
Separado/Divorciado	17	4,49
Viúvo	15	3,96
Escolaridade		
Analfabeto	20	5,25
Ensino Fundamental	26	6,82
Ensino Médio Completo	153	40,16
Ensino Médio Incompleto	5	1,31
Ensino Superior Completo	110	28,87
Ensino Superior Incompleto	67	17,59
Etnia		
Pardo	223	62,29
Branco	71	19,83
Preto	63	17,60
Indígena	1	0,28
Situação econômica		
Empregado	288	75,99
Desempregado	46	12,14
Aposentado	12	3,17
Estudante	33	8,71
Renda Familiar^§^		
Até 1 salário-mínimo	83	21,96
1 a 2 salários-mínimos	135	35,71
Mais de 2 salários-mínimos	160	42,33
**Clínico-epidemiológicos**			
Antecedentes familiares de doenças crônicas		
Não	178	46,84
Sim	202	53,16
Comorbidades		
Não	315	83,11
Sim	64	16,89
Doenças respiratórias		
Não	268	70,53
Sim	112	29,47
Internação hospitalar		
Não	371	98,15
Sim	7	1,85
Histórico de transtornos mentais		
Não	328	87
Sim	49	13
**Eventos estressantes relacionados à pandemia**			
Perda familiar pela COVID-19		
Não	302	79,26
Sim	78	20,53
Perda familiar relacionada a outras doenças		
Não	259	68,88
Sim	117	31,12
Medo de contrair COVID-19 novamente		
Não	192	50,66
Sim	187	49,34
Trabalhava antes da pandemia		
Não	108	28,57
Sim	270	71,43
Situação financeira atual		
Não contribui com a renda familiar	39	10,34
Perdeu a renda durante a pandemia	14	3,71
Contribui com a renda familiar	217	57,56
Responsável pela renda familiar	107	28,38

*DP = Desvio-padrão; ^†^n = Número absoluto; ^‡^% = Frequência relativa; ^§^Salário-mínimo vigente = R$ 1.320,00, Brasil, 2023

Ao analisar os escores da Escala Hospitalar de Ansiedade e Depressão (HADS), verificou-se que 53,54% (n = 204) dos participantes apresentaram perfil improvável de ansiedade, 25,98% (n = 99) perfil de possível ansiedade e 20,47% (n = 78) perfil de provável ansiedade. Em relação à depressão, os resultados mostraram que 77,69% (n = 296) são casos improváveis, 16,01% (n = 61) são casos possíveis e 6,30% (n = 24) são casos prováveis (Tabela 2). Para fins de análise estatística inferencial, as categorias “possível” e “provável” foram agrupadas, constituindo uma variável dicotômica (improvável versus possível/provável). Essa estratégia foi adotada para atender aos pressupostos estatísticos e aumentar a robustez das análises. Com isso, a prevalência de ansiedade possível/provável foi de 46,45%, enquanto a prevalência de depressão possível/provável foi de 22,31%.

Observou-se associação estatisticamente significativa entre ansiedade e as variáveis sexo, situação econômica, renda familiar, antecedentes familiares, presença de doenças respiratórias, histórico de transtorno mental, medo de reinfecção por COVID-19 e recebimento de auxílio governamental (p < 0,05) (Tabela 3). 

Quanto à depressão, verificou-se associação estatisticamente significativa com as variáveis sexo, renda familiar, antecedentes familiares, presença de comorbidades, histórico de transtorno mental, perda familiar por COVID-19, medo de reinfecção por COVID-19 e recebimento de auxílio governamental (p < 0,05) (Tabela 3).

**Tabela 2:** Análise da Escala Hospitalar de Ansiedade e Depressão entre pacientes acometidos pela COVID-19. Petrolina, PE, Brasil, 2023

Variáveis	n*	%^†^
Ansiedade		
Improvável	204	53,54
Possível	99	25,98
Provável	78	20,47
**Depressão**		
Improvável	296	77,69
Possível	61	16,01
Provável	24	6,30

*n = Número absoluto; ^†^% = Frequência relativa

**Tabela 3:** Associação da ansiedade e da depressão com características sociodemográficas, clínico-epidemiológicas e eventos estressantes relacionados à pandemia entre pacientes acometidos pela COVID-19. Petrolina, PE, Brasil, 2023

Variáveis	Ansiedade				p-valor	Depressão				p-valor
	Improvável			Possível/ provável		Improvável		Possível/ provável		
	n*	%^†^	n*	%^†^		n*	%^†^	n*	%^†^	
**Sexo**										
Masculino	94	46.1	37	20.9	<0.001^‡^	116	39.2	15	17.7	<0.001^‡^
Feminino	110	53.9	140	79.1		180	60.8	70	82.4
**Estado civil (casado/união estável)**										
Não casado/união estável	101	49.5	95	53.7	0.417^‡^	148	50.0	48	56.5	0.293^‡^
Casado/união estável	103	50.5	82	46.3		148	50.0	37	43.5
**Escolaridade (baixa)**										
≥ Ensino Médio	177	86.8	158	89.3	0.455^‡^	260	87.8	75	88.2	0.921^§^
≤ Ensino Fundamental	27	13.2	19	10.7		36	12.2	10	11.8
**Etnia**										
Branco	55	27.0	39	22.0	0.266^‡^	76	25.7	18	21.2	0.396^‡^
Não branco	149	73.0	138	78.0		220	74.3	67	78.8
**Situação econômica**										
Não empregado	36	17.7	57	32.2	0.001^‡^	71	24.0	22	25.9	0.720^‡^
Empregado	168	82.4	120	67.8		225	76.0	63	74.1
**Renda familiar (até 1 SM)^||^**										
>1 salário mínimo	169	82.8	129	72.9	0.019^‡^	241	81.4	57	67.1	0.005^‡^
≤1 salário mínimo	35	17.2	48	27.1		55	18.6	28	32.9
**Antecedentes familiares**										
Não	110	53.9	68	38.6	0.003^‡^	149	50.5	29	34.1	0.008^‡^
Sim	94	46.1	108	61.4		146	49.5	56	65.9
**Comorbidades**										
Não	174	85.3	141	80.6	0.221^‡^	252	85.1	63	75.9	0.047^‡^
Sim	30	14.7	34	19.4		44	14.9	20	24.1
**Doenças respiratórias**										
Não	154	75.5	114	64.8	0.022^‡^	213	72.2	55	64.7	0.182^‡^
Sim	50	24.5	62	35.2		82	27.8	30	35.3
**Internação hospitalar**										
Não	199	98.0	172	98.3	0.854^§^	287	97.6	84	100.0	0.356^§^
Sim	4	2.0	3	1.7		7	2.4	0	0.0
**Histórico de transtorno mental**										
Não	192	95.1	136	77.7	<0.001^‡^	265	90.4	63	75.0	<0.001^‡^
Sim	10	5.0	39	22.3		28	9.6	21	25.0
**Perda familiar por COVID-19**										
Não	166	81.8	136	76.8	0.235^‡^	242	81.8	60	71.4	0.039^‡^
Sim	37	18.2	41	23.2		54	18.2	24	28.6
**Perda familiar por outras doenças**										
Não	134	66.7	125	71.4	0.320^‡^	205	70.2	54	64.3	0.302^‡^
Sim	67	33.3	50	28.6		87	29.8	30	35.7
**Medo de contrair COVI-19 novamente**										
Não	130	64.4	62	35.0	<0.001^‡^	168	57.1	24	28.2	<0.001^‡^
Sim	72	35.6	115	65.0		126	42.9	61	71.8
**Trabalhou durante a pandemia**										
Não	55	27.2	53	30.1	0.536^‡^	82	28.0	26	30.6	0.640^‡^
Sim	147	72.8	123	69.9		211	72.0	59	69.4
**Auxílio governamental**										
Não	184	92.0	140	81.9	0.003^‡^	260	89.7	64	79.0	0.011^‡^
Sim	16	8.0	31	18.1		30	10.3	17	21.0
**Responsável pela renda familiar**										
Não	149	73.0	125	70.6	0.600^‡^	218	73.7	56	65.9	0.160^‡^
Sim	55	27.0	52	29.4		78	26.4	29	34.1

*n = Número absoluto; ^†^% = Frequência relativa; ^‡^Qui-quadrado; ^§^Exato de Fisher; ^||^Salário-mínimo vigente = R$ 1.320,00, Brasil, 2023

Os fatores associados à ansiedade, evidenciados por meio dos modelos de regressão, indicaram que o medo de contrair COVID-19 novamente esteve associado a maiores chances de ansiedade (possível/provável) (OR = 3,08; IC95% 1,95-4,88; p < 0,001), assim como o histórico prévio de transtorno mental (OR = 5,56; IC95% 2,42-12,76; p < 0,001). A situação econômica apresentou associação inversa, com menor chance de ansiedade entre participantes empregados, caracterizando um efeito protetor, com redução de aproximadamente 46% na chance de ansiedade em relação às demais categorias (OR = 0,54; IC95% 0,31-0,93; p = 0,027). Também se observou aumento da chance de ansiedade na presença de antecedentes familiares de doenças crônicas (OR = 1,76; IC95% 1,11-2,79; p = 0,016) (Tabela 4). 

No que se refere à depressão, evidenciou-se associação com o medo de contrair COVID-19 (possível/provável), com OR = 3,24 (IC95% 1,85-5,69; p < 0,001). Além disso, apresentaram maiores chances de depressão os participantes com histórico de transtorno mental (OR = 2,80; IC95% 1,40-5,58; p = 0,003), renda familiar de até um salário mínimo (OR = 2,18; IC95% 1,21-3,93; p = 0,009), antecedentes familiares de doenças crônicas (OR = 1,81; IC95% 1,04-3,13; p = 0,035) e presença de comorbidades (OR = 1,97; IC95% 1,03-3,78; p = 0,041), conforme apresentado na Tabela 4.

**Tabela 4:** Modelos de regressão logística binária dos fatores associados à ansiedade e à depressão entre pacientes acometidos pela COVID-19. Petrolina, PE, Brasil, 2023

Variável	Ansiedade				Depressão			
	OR* ajustado	IC95%^†^		p-valor	OR* ajustado	IC95%^†^		p-valor
Medo de contrair COVID-19 novamente								
Sim vs^‡^ Não	3,08	1,95	4,88	<0,001	3,24	1,85	5,69	<0,001
Histórico de transtorno mental								
Sim vs^‡^ Não	5,56	2,42	12,76	<0,001	2,80	1,40	5,58	0,003
Situação econômica								
Empregado	0,54	0,31	0,93	0,027				
Antecedentes familiares de doenças crônicas								
Sim vs^‡^ Não	1,76	1,11	2,79	0,016	1,81	1,04	3,13	0,035
Renda familiar^§^ de até 1 salário-mínimo								
≤ 1 salário-mínimo vs^‡^ superior a 1 salário-mínimo	1,69	0,96	2,97	0,071	2,18	1,21	3,93	0,009
Tempo desde a COVID-19 (meses)								
Aumento de 1 mês	0,98	0,95	1,01	0,133				
Comorbidades								
Sim vs^‡^ Não					1,97	1,03	3,78	0,041

*OR - *Odds Ratio*; ^†^IC95% - Intervalo de Confiança de 95%; ^‡^vs - *Versus*; teste Hosmer-Lemeshow de ajuste do modelo: Ansiedade: chi²(8) = 2,12 e p = 0,9773; Depressão: chi²(8) = 13,92 e p = 0,0839; ^§^Salário-mínimo vigente = R$ 1.320,00, Brasil, 2023

## Discussão

Este estudo identificou elevada prevalência de sintomas ansiosos e depressivos em indivíduos avaliados após infecção por SARS-CoV-2, indicando persistência de alterações na saúde mental no período pós-agudo da COVID-19. Esses desfechos estiveram associados a características sociodemográficas, clínicas e psicossociais, com destaque para o sexo feminino, a vulnerabilidade socioeconômica e o medo persistente de reinfecção.

A prevalência de ansiedade observada foi superior ao aumento global estimado de 25% no primeiro ano da pandemia de COVID-19^3^, bem como às estimativas descritas para a população brasileira em geral antes e durante a pandemia, que indicam prevalência aproximada de 14,3% para ansiedade e entre 4% e 5% para depressão^15^ ^-^ ^16^. Esse achado constitui um aspecto relevante do estudo, ao evidenciar que indivíduos no período pós-COVID-19 mantiveram risco aumentado para sofrimento psíquico quando comparados à população geral. Em comparação com a coorte brasileira de pacientes hospitalizados que utilizou o mesmo instrumento, as prevalências encontradas diferiram^10^, o que pode estar relacionado a diferenças contextuais e metodológicas. A presente amostra foi composta predominantemente por casos leves, acompanhados em contexto domiciliar, o que pode ter favorecido a persistência de sintomas psicológicos menos intensos clinicamente, porém duradouros.

Os resultados mostraram-se coerentes com evidências longitudinais internacionais. Estudos conduzidos com sobreviventes da COVID-19 acompanhados após tratamento hospitalar identificaram prevalências mais elevadas de ansiedade (42%) e depressão (31%)^17^, sugerindo que a gravidade clínica inicial pode modular esses desfechos. Por outro lado, coortes com seguimento prolongado demonstraram que, embora os sintomas depressivos tendessem a reduzir ao longo do tempo, os níveis de ansiedade permaneceram estáveis, indicando que a ansiedade apresenta trajetória mais persistente que a depressão no período pós-COVID-19^18^. Nesse contexto, evidências recentes apontaram que ansiedade e depressão tendem a ocorrer de forma concomitante e atuam como fatores preditivos da condição pós-COVID-19, reforçando a hipótese de que o sofrimento psíquico integra o espectro clínico da COVID longa^5^.

Além das elevadas prevalências, os modelos de regressão logística evidenciaram que o medo de um novo contágio por COVID-19 e o histórico prévio de transtorno mental foram os principais fatores associados tanto à ansiedade quanto à depressão, aumentando em aproximadamente três vezes as chances desses desfechos. Adicionalmente, renda familiar de até um salário mínimo, antecedentes familiares de doenças crônicas e presença de comorbidades associaram-se a maior probabilidade de depressão, enquanto estar empregado mostrou-se fator protetor para a ansiedade.

Observou-se maior prevalência de ansiedade e depressão entre mulheres, resultado semelhante ao descrito em estudos nacionais^10^ ^,^ ^17^ e internacionais^19^. Essa maior vulnerabilidade da população feminina ao adoecimento por transtornos mentais, em comparação aos homens, também foi identificada na população brasileira durante a pandemia de COVID-19^17^. Esse achado está em consonância com relatórios globais que apontam risco entre 1,5 e 2 vezes maior para transtornos mentais entre mulheres^3^. Ademais, metanálise recente envolvendo sobreviventes da COVID-19 identificou maior chance de ansiedade (OR=2,1) e depressão (OR=1,8) entre mulheres, reforçando que desigualdades de gênero persistiram no contexto pós-pandêmico^19^. A maior vulnerabilidade feminina foi atribuída à sobrecarga de atividades domésticas e de cuidado, à insegurança econômica e ao impacto psicossocial ampliado durante a pandemia^19^.

No que se refere aos determinantes socioeconômicos, os achados evidenciaram que estar empregado exerceu efeito protetor para a ansiedade, enquanto a baixa renda familiar aumentou significativamente a chance de depressão. Esses resultados estiveram alinhados a estudos longitudinais recentes que demonstraram que o desemprego elevou em até 2,4 vezes a chance de ansiedade e que a baixa renda duplicou o risco de depressão em seguimento prolongado após a COVID-19^20^. De forma semelhante, estudo sobre trajetórias pós-pandêmicas identificou que renda familiar ≤1 salário mínimo aumentou em 2,1 vezes a chance de depressão, enquanto o emprego manteve efeito protetor em torno de 40%, corroborando o padrão observado nesta investigação^21^.

O histórico prévio de transtorno mental destacou-se como um dos fatores mais fortemente associados à ansiedade e à depressão, resultado consistente com análises longitudinais de três anos, que apontaram elevada recorrência ou persistência de transtornos mentais após a COVID-19 em indivíduos com vulnerabilidade psiquiátrica prévia^22^. Evidências adicionais indicaram que indivíduos com transtornos mentais prévios apresentaram risco até cinco vezes maior de desenvolver sintomas ansiosos e depressivos no contexto da COVID longa^23^. 

De modo semelhante, a associação entre doenças crônicas, comorbidades e sintomas depressivos observada neste estudo mostrou-se coerente com metanálises que apontaram maior chance de ansiedade e depressão entre indivíduos com condições crônicas, com OR variando entre 1,7 e 2,0, possivelmente mediado por mecanismos de neuroinflamação persistente^5^. Estudos também demonstraram que a presença de comorbidades quase duplicou a chance de depressão (OR=1,9) e que doenças respiratórias aumentaram o risco de ansiedade (OR=1,6), reforçando a interação entre adoecimento físico e sofrimento psíquico no período pós-COVID-19^24^.

Os modelos múltiplos mostraram que pacientes com histórico familiar de comorbidades e com histórico pessoal de transtorno mental apresentaram maior probabilidade de ansiedade e depressão, em consonância com estudos epidemiológicos que apontam o antecedente psiquiátrico como importante fator de vulnerabilidade para o desenvolvimento ou agravamento de distúrbios psicológicos após a infecção^5^ ^,^ ^22^ ^-^ ^23^. Adicionalmente, a presença de comorbidades clínicas quase quadruplicou a chance de depressão, reforçando o papel do adoecimento crônico na sobrecarga emocional desses pacientes.

Além disso, no presente estudo, o medo de se contaminar novamente por SARS-CoV-2 esteve fortemente associado tanto à ansiedade quanto à depressão. Pesquisa realizada no estado de Mato Grosso do Sul verificou que 64% dos participantes relataram medo de contrair a doença novamente^24^, condição que tende a aumentar a carga emocional e física, favorecendo o sofrimento psicológico. Estudos internacionais evidenciaram que o medo de contrair COVID-19 novamente emergiu como o principal preditor de ansiedade e depressão, achado consistente com investigações sobre trajetórias de saúde mental que identificaram aumento de chance entre 2,8 e 3,5 para ambos os desfechos associados ao medo persistente^21^. Metanálise específica sobre fatores psicológicos na COVID longa reforçou esse resultado ao demonstrar que o medo de reinfecção aumentou em aproximadamente três vezes a chance de ansiedade, configurando-se como um dos determinantes mais robustos do sofrimento psíquico prolongado^5^.

As principais limitações do presente estudo foram o delineamento transversal, que impossibilitou inferências causais, e o predomínio de casos leves, o que limita a generalização dos resultados para populações com maior gravidade clínica. Além disso, a escassez de estudos nacionais com delineamento semelhante restringiu comparações mais amplas. 

Apesar dessas limitações, este estudo contribuiu para o avanço do conhecimento científico nas áreas da saúde e da enfermagem ao produzir evidências nacionais sobre a prevalência e os fatores associados à ansiedade e à depressão no período pós-COVID-19, em uma população predominantemente composta por casos leves acompanhados em contexto não hospitalar. Ao integrar variáveis sociodemográficas, clínicas e psicossociais em modelos analíticos ajustados, os achados ampliaram a compreensão dos determinantes do sofrimento psíquico após a infecção por SARS-CoV-2. Essas evidências fortalecem a base científica para o desenvolvimento de estratégias de rastreamento, acompanhamento e cuidado integral em saúde mental, com repercussões diretas para a prática assistencial, o planejamento em saúde e a produção de conhecimento em enfermagem.

## Conclusão 

O estudo identificou elevada prevalência de ansiedade entre sobreviventes da COVID-19, com maior probabilidade entre pessoas com medo de novo contágio, histórico pessoal de transtorno mental, antecedentes familiares de doenças crônicas, baixa renda e situação de desemprego, enquanto estar empregado mostrou efeito protetor. A depressão apresentou prevalência inferior à observada para ansiedade, mas esteve associada principalmente ao medo de reinfecção, ao histórico de transtorno mental, à baixa renda, aos antecedentes familiares de doenças crônicas e à presença de comorbidades clínicas. 

Esses achados reforçam a necessidade de estratégias de rastreamento sistemático de sintomas ansiosos e depressivos na população pós-COVID-19, com enfoque prioritário em grupos socialmente vulneráveis e em indivíduos com antecedentes psiquiátricos. Ademais, destacam a importância da oferta de intervenções psicossociais que possam reduzir o impacto da COVID-19 na saúde mental.

## Data Availability

Todos os dados gerados ou analisados durante este estudo estão incluídos neste artigo publicado.

# Translation: Prevalencia y factores asociados a la ansiedad y a la depresión post COVID-19^*^

**DOI:** 10.1590/1518-8345.8173.4914

**Authors:** Iasmin Daniele de Araújo, Isabella Joyce Silva de Almeida Carvalho, Lusineide Carmo Andrade de Lacerda, Luciana Pessoa Maciel Diniz, Flávia Emília Cavalcante Valença Fernandes, Roxana Braga de Andrade Teles

**Affiliations:** 1 Universidade de Pernambuco, Campus Petrolina, Petrolina, PE, Brasil.; 2 Bolsista da Fundação de Amparo à Ciência e Tecnologia do Estado de Pernambuco (FACEPE), Brasil.

**Keywords:** Ansiedad, Depresión, COVID-19, Síndrome Post Agudo de COVID-19, Salud Mental, Factores de Riesgo

## Abstract

**Objetivo:**

estimar la prevalencia y los factores asociados a la ansiedad y a la depresión en pacientes que se están recuperando de COVID-19.

**Método:**

estudio transversal realizado con 381 pacientes cuatro meses después de la infección por SARS-COV-2. Se recolectaron datos sociodemográficos y clínicoepidemiológicos, al igual que diversa información sobre la incidencia de sucesos estresantes relacionados con la pandemia de COVID-19, por medio de un cuestionario y aplicando la Escala de Ansiedad y Depresión Hospitalaria. Se realizaron análisis descriptivos e inferenciales con regresión logística binaria para identificar los factores asociados.

**Resultados:**

los valores de prevalencia de ansiedad y depresión posible y probable fueron 46,45% y 22,31%, respectivamente. El temor de volver a contraer COVID-19, antecedentes previos de trastornos mentales y antecedentes familiares de enfermedades crónicas estuvieron asociados a mayores probabilidades de sufrir ansiedad y depresión. Ingresos familiares de hasta un salario mínimo y presencia de comorbilidades estuvieron asociados a la depresión, mientras que tener empleo se erigió como un factor de protección en el caso de la ansiedad.

**Conclusión:**

se observó elevada prevalencia de ansiedad y depresión durante el período post COVID-19, asociada con factores psicosociales, clínicos y socioeconómicos, evidenciando así la necesidad de implementar estrategias de detección y atención de la salud mental en esta población.

## Destacados

**(1)** Elevada prevalencia de ansiedad en pacientes recuperados de COVID-19. **(2)** Factores como sexo, situación económica, fallecimientos y temor de contraer COVID-19 influenciaron la salud emocional. **(3)** Factores sociales/clínicos y el estrés asociado a la pandemia estuvieron relacionados con la ansiedad y la depresión. **(4)** Implementar intervenciones y realizar un diagnóstico temprano son medidas esenciales en la atención psicológica después de COVID-19.

## Introducción

Causada por el virus SARS-CoV-2, la epidemia de COVID-19 fue declarada pandemia en marzo de 2020, configurándose como uno de los principales desafíos sanitarios del siglo XXI^1^. Actualmente hay más de 700 millones de casos confirmados a nivel global^2^. Aunque los esfuerzos iniciales se hayan concentrado en reducir la morbimortalidad asociada a la fase aguda de la enfermedad, cada vez más evidencias indican la persistencia de manifestaciones físicas y psicológicas después de la infección, caracterizando el período post COVID-19^3^.

La persistencia de los síntomas relacionados con COVID-19 puede perdurar por semanas o meses. Por tratarse de una enfermedad nueva con limitada información sobre sus manifestaciones crónicas, el cuadro clínico a largo plazo inicialmente recibió menos atención en comparación con los aspectos clínicos de la fase aguda^4^. Sin embargo, algunos de los casos demostraron ser clínicamente relevantes, puesto que pacientes con cuadros leves o graves comenzaron a presentar síntomas y secuelas a largo plazo, incluso después de al menos dos meses de contraer la infección, con afectación de diversos sistemas orgánicos e incluso con manifestaciones psicológicas^5^. Estas manifestaciones prolongadas de COVID-19 ejercen efectos negativos sobre la calidad de vida, lo que dificulta la recuperación y el regreso a las actividades diarias^6^.

El debate sobre las complicaciones físicas en los pacientes durante el período post COVID-19 es extenso; en cambio, las consecuencias para la salud mental a largo plazo todavía no han recibido la atención necesaria^5^. Evidencias recientes demuestran que la infección por SARS-CoV-2 puede causar alteraciones en los circuitos neuroinmunes, aumentando así el riesgo de que surjan y/o se agraven comorbilidades en las personas afectadas. Además, el estrés psicosocial y la forma grave de la enfermedad son factores de riesgo para el desarrollo de señales y síntomas de trastornos psicológicos^7^.

La infección por COVID-19 está asociada a una sobrecarga mental resultado de sucesos estresantes, inseguridad, ansiedad y miedo, lo que contribuye al desarrollo de trastornos psicológicos^8^. Estos trastornos pueden entenderse como manifestaciones de los sucesos estresantes vividos por las víctimas de COVID-19, ocasionando síntomas y señales de ansiedad y depresión en aproximadamente el 30% al 40% de los pacientes durante el período posterior a la infección^9^. Un estudio transversal realizado con 70 participantes evidenció que apenas ocho (11,4%) no presentaban síntomas relacionados con la depresión, que varió de leve a moderada. En relación a la ansiedad, se observó que 18 individuos (25,7%) presentaron síntomas asociados a COVID-19 después de padecer la enfermedad^7^.

Considerando la elevada prevalencia de ansiedad y depresión durante el período post COVID-19^10^, al igual que sus repercusiones físicas, mentales y sociales que contribuyen a ausencias laborales, a la pérdida de productividad y a una reducción en la calidad de vida, además de la escasez de estudios encontrada en la literatura, se hace evidente la necesidad de realizar trabajos de investigación sobre cambios en el estado mental entre sobrevivientes de COVID-19. En este contexto, el objetivo de este estudio es estimar la prevalencia y los factores asociados a la ansiedad y a la depresión en pacientes que se están recuperando de COVID-19.

## Método

### Diseño del estudio

Estudio analítico de tipo transversal basado en las pautas indicadas en *Strengthening the Reporting of Observational Studies in Epidemiology*(STROBE)^11^.

### Lugar y período del estudio

El estudio se realizó entre septiembre de 2022 y julio de 2023 en un municipio de Petrolina (Pernambuco), en el Hospital Universitario de la *Universidade Federal do Vale do São Francisco* (HU-UNIVASF) y en el Centro para la Detección de COVID-19.

### Población

La población del estudio estuvo conformada por pacientes con antecedentes de infección por COVID-19, 4 meses después de diagnosticada la enfermedad.

### Criterios de selección

Las personas incluidas en la muestra tenían al menos 18 años de edad, eran de ambos sexos y su diagnóstico de COVID-19 se había confirmado por medio de la prueba de Reacción en Cadena de la Polimerasa (*Polymerase Chain Reaction*, PCR) (o RT-PCR) para el virus SARS-CoV-2, con un mínimo de cuatro meses después de la infección. Se excluyó a los individuos con déficits cognitivos que les imposibilitaran comprender los instrumentos de la investigación, al igual que a las personas que no respondieron los instrumentos en su totalidad, lo que inviabilizó el procesamiento y análisis de los datos.

### Participantes

El cálculo muestral se realizó sobre la base de la cantidad estimada de personas afectadas por COVID-19 durante el año 2022 en el municipio de Petrolina, considerando un nivel de confianza del 95%. El tamaño de la muestra se calculó utilizando la herramienta OpenEpi (http://www.OpenEpi.com), resultando una muestra compuesta por 381 individuos.

### Variables del estudio

El resultado primario del estudio fue la prevalencia de ansiedad y depresión. Las variables independientes incluyeron características sociodemográficas y clínicoepidemiológicas, al igual que sucesos estresantes relacionados con la pandemia de COVID-19.

### Instrumentos utilizados para recolectar los datos

Se empleó un cuestionario estructurado elaborado por los investigadores para recolectar datos sociodemográficos y clínicoepidemiológicos, además de la incidencia de sucesos estresantes relacionados con la pandemia de COVID-19, como desempleo, luto y cambios en la estructura familiar. También se utilizó la Escala de Ansiedad y Depresión Hospitalaria (*Hospital Anxiety and Depression Scale*, HADS). Fue posible autoaplicar ambos instrumentos, con la debida supervisión de la investigadora. En el caso de participantes con dificultades para leer, la entrevistadora leyó íntegramente las preguntas junto con las respectivas opciones de respuesta.

HADS es un instrumento adaptado y validado para ser usado en Brasil y presenta buenos parámetros para detectar síntomas de ansiedad y depresión^12^. Inicialmente desarrollada con el fin de evaluar estos síntomas en pacientes internados en hospitales clínicos no psiquiátricos, posteriormente también comenzó a usarse esta escala en individuos no hospitalizados y sin comorbilidades^13^.

La escala está conformada por 14 ítems distribuidos en dos subescalas: Ansiedad (HADS-A) y Depresión (HADS-D), con siete ítems cada una. Cada ítem ofrece cuatro opciones de respuesta y la puntuación varía de cero a tres, totalizando 21 puntos para cada subescala. La puntuación final se calcula sumando los valores indicados por los participantes. Además, las puntuaciones se pueden categorizar de acuerdo con el grado en el que se manifiesta la sintomatología (normal, leve, moderado y grave). En este estudio se consideran casos posibles, probables y graves aquellos con puntuaciones superiores a los 8, 11 y 15 puntos, respectivamente^14^.

### Recolección de datos

Para recolectar los datos, el primer paso fue realizar un análisis de las historias clínicas de los pacientes del HU-UNIVASF y de los datos del Centro para la Detección de COVID-19 (puestos a disposición por la Secretaría Municipal de Salud), con el fin de seleccionar a los individuos que cumplían con los criterios de elegibilidad del estudio. Subsiguientemente, se invitó a las personas elegidas a participar en la investigación. Cuando aceptaron, se realizaron entrevistas semiestructuradas individuales dirigidas por la investigadora. Estas entrevistas tuvieron lugar en el hogar de cada participante o en un ambiente privado y duraron una media de 20 minutos.

### Análisis de los datos

Las variables se tabularon en Microsoft Office Excel^®^ 2016 para registrar y codificar los datos. Posteriormente, se exportó toda la información al programa estadístico Stata (versión 16.0) para realizar el análisis descriptivo y de asociación entre las variables. En el análisis descriptivo se calcularon frecuencias absolutas y relativas para las variables categóricas, mientras que se tomaron medidas de tendencia central y dispersión para las numéricas.

El análisis inferencial de los factores asociados a la ansiedad y a la depresión se realizó por medio de regresión logística binaria. Para esta etapa, las variables de desenlace se dicotomizaron en presencia de ansiedad y presencia de depresión (casos posibles o probables: puntuaciones ≥8 puntos *versus* casos poco probables), conforme a la clasificación obtenida en HADS. Como primer paso, se realizaron análisis bivariados entre cada desenlace y las variables sociodemográficas, clínicoepidemiológicas y las relacionadas con sucesos estresantes, utilizando las pruebas chi-cuadrado de Pearson o exacta de Fisher y adoptando valores p <0,20 como criterio para seleccionar las variables con posibilidades de ser incluidas en los modelos múltiples.

Subsiguientemente, se realizó un modelado por regresión logística binaria con selección automática del tipo *stepwise forward*, incluyendo en el modelo inicial todas las variables con p <0,20 en el análisis bivariado. Las variables que permanecieron en el modelo final fueron las asociadas al desenlace con un nivel de significancia del 5%, con una estimación de las razones de probabilidades (*Odds Ratio*, OR) ajustadas y los respectivos intervalos de confianza del 95%. El ajuste y el desempeño de los modelos se evaluaron por medio de la prueba de Hosmer-Lemeshow.

### Aspectos éticos

La investigación fue aprobada por el Comité de Ética en Investigación del Centro Integrado de Salud Amaury de Medeiros (*Comitê de Ética em Pesquisa do Centro Integrado de Saúde Amaury de Medeiros*, CEP-CISAM/UPE), con número de opinión 5.760.468 y registrado en el Certificado de Presentación para Apreciación Ética (CAAE) número 57873222.1.0000.5191.

## Resultados

Los participantes del estudio fueron 381 personas con una media de edad de 36,72 años (±13,14) y tiempo medio posterior a la infección por COVID-19 de 15,19 meses (±8,73). Se observó predominio del sexo femenino (65,62%), de personas autodeclaradas como de piel morena (62,29%), solteras (42,74%) y con Escuela Secundaria completa (40,16%). En relación a la situación económica, el 75,99% de los participantes estaba trabajando, con ingresos familiares superiores a 2 salarios mínimos en el 42,33% de los casos (Tabla 1).

La mayoría declaró antecedentes familiares de enfermedades crónicas (53,16%) y no presentaba antecedentes personales de trastornos mentales (87%) ni de enfermedades respiratorias (70,53%) u otras comorbilidades asociadas (83,11%). En referencia al tratamiento de COVID-19, el 98,15% de los pacientes lo siguieron en sus casas, puesto que se los había clasificado como casos leves (Tabla 1).

En lo que se refiere a los sucesos estresantes relacionados con la pandemia, se observó que la mayoría de los participantes (79,26%) no sufrió ninguna pérdida debido a COVID-19 ni a otras enfermedades (70,47%). Cuando se les preguntó sobre el temor a volver a infectarse de COVID-19, el 50,66% asintió. Además, la mayoría trabajó durante la pandemia (71,43%), y el 57,56% aportaba al sustento de la familia (Tabla 1).

**Tabla 1:** Datos sociodemográficos y clínicoepidemiológicos y sucesos estresantes relacionados con la pandemia entre pacientes afectados por COVID-19. Petrolina, PE, Brasil, 2023

Variables	Media	DE*
Edad (años)	36,72	13,14
Tiempo desde diagnóstico de COVID-19 (meses)	15,19	8,73
**Sociodemográficas**			
Variables	n^†^	%^‡^
Sexo		
Masculino	131	34,38
Femenino	250	65,62
Estado civil		
Soltero(a)	162	42,74
Unión estable	41	10,82
Casado(a)	144	37,99
Separado(a)/Divorciado(a)	17	4,49
Viudo(a)	15	3,96
Nivel de estudios		
Analfabeto(a)	20	5,25
Escuela Primaria	26	6,82
Escuela Secundaria completa	153	40,16
Escuela Secundaria incompleta	5	1,31
Educación Superior completa	110	28,87
Educación Superior incompleta	67	17,59
Etnia/Color de piel		
Morena	223	62,29
Blanca	71	19,83
Negra	63	17,60
Indígena	1	0,28
Situación económica		
Empleado	288	75,99
Desempleado	46	12,14
Jubilado	12	3,17
Estudiante	33	8,71
Ingresos familiares^§^		
Hasta 1 salario mínimo	83	21,96
De 1 a 2 salarios mínimos	135	35,71
Más de 2 salarios mínimos	160	42,33
**Clinicoepidemiológicas**			
Antecedentes familiares de enfermedades crónicas		
No	178	46,84
Sí	202	53,16
Comorbilidades		
No	315	83,11
Sí	64	16,89
Enfermedades respiratorias		
No	268	70,53
Sí	112	29,47
Internación en hospital		
No	371	98,15
Sí	7	1,85
Antecedentes de trastornos mentales		
No	328	87
Sí	49	13
**Sucesos estresantes relacionados con la pandemia**			
Fallecimiento de familiar(es) a raíz de COVID-19		
No	302	79,26
Sí	78	20,53
Fallecimiento de familiar(es) relacionado con otras enfermedades			
No	259	68,88
Sí	117	31,12
Temor de volver a contraer COVID-19		
No	192	50,66
Sí	187	49,34
Trabajaba antes de la pandemia		
No	108	28,57
Sí	270	71,43
Situación financiera actual		
No aporta a los ingresos familiares	39	10,34
Perdió sus ingresos durante la pandemia	14	3,71
Aporta a los ingresos familiares	217	57,56
Es el sustento económico de la familia	107	28,38

*DE = Desviación Estándar; ^†^n = Cantidad absoluta; ^‡^% = Frecuencia relativa; ^§^Salario mínimo vigente = R$ 1320,00, Brasil, 2023

Al analizar las puntuaciones de la Escala de Ansiedad y Depresión Hospitalaria (*Hospital Anxiety and Depression Scale*, HADS), se verificó que el 53,54% (n=204), el 25,98% (n=99) y el 20,47% (n=78) de los participantes presentaron los perfiles de ansiedad poco probable, posible y probable, respectivamente. Con respecto a la depresión, los resultados indicaron que el 77,69% (n=296), el 16,01% (n=61) y el 6,30% (n=24) eran casos poco probables, posibles y probables, respectivamente (Tabla 2). A los propósitos del análisis estadístico inferencial, se agruparon las categorías “Posible” y “Probable”, constituyendo una variable dicotómica (Poco probable versus Posible/Probable). Se adoptó esta estrategia para respetar las presunciones estadísticas y mejorar la robustez de los análisis. En consecuencia, la prevalencia de ansiedad posible/probable fue del 46,45%, mientras que la de depresión posible/probable correspondió al 22,31%.

Se observó una asociación estadísticamente significativa entre ansiedad y las siguientes variables: sexo, situación económica, ingresos familiares, antecedentes familiares, presencia de enfermedades respiratorias, antecedentes de trastornos mentales, temor de volver a infectarse de COVID-19 y recibir asistencia del gobierno (p<0,05) (Tabla 3).

En cuanto a la depresión, se verificó una asociación estadísticamente significativa con estas variables: sexo, ingresos familiares, antecedentes familiares, presencia de comorbilidades, antecedentes de trastornos mentales, fallecimientos en la familia por causa de COVID-19, temor de volver a infectarse de COVID-19 y recibir asistencia del gobierno (p<0,05) (Tabla 3).

**Tabla 2:** Análisis de la Escala de Ansiedad y Depresión Hospitalaria entre pacientes afectados por COVID-19. Petrolina, PE, Brasil, 2023

Variables	n*	%^†^
**Ansiedad**		
Poco probable	204	53,54
Posible	99	25,98
Probable	78	20,47
**Depresión**		
Poco probable	296	77,69
Posible	61	16,01
Probable	24	6,30

*n = Cantidad absoluta; ^†^% = Frecuencia relativa

**Tabla 3:** Asociación de ansiedad y depresión con características sociodemográficas/clínicoepidemiológicas y sucesos estresantes relacionados con la pandemia entre pacientes afectados por COVID-19. Petrolina, PE, Brasil, 2023

Variables	Ansiedad				Valor p	Depresión				Valor p
	Poco probable			Posible/Probable		Poco probable		Posible/Probable		
	n*	%^†^	n*	%^†^		n*	%^†^	n*	%^†^	
**Sexo**										
Masculino	94	46.1	37	20.9	<0.001^‡^	116	39.2	15	17.7	<0.001^‡^
Feminino	110	53.9	140	79.1		180	60.8	70	82.4
**Estado civil (casado/unión estable)**										
No casado/unión estable	101	49.5	95	53.7	0.417^‡^	148	50.0	48	56.5	0.293^‡^
Casado/unión estable	103	50.5	82	46.3		148	50.0	37	43.5
**Nivel de estudios (bajo)**										
≥ Escuela Secundaria	177	86.8	158	89.3	0.455^‡^	260	87.8	75	88.2	0.921^§^
≤ Escuela Primaria	27	13.2	19	10.7		36	12.2	10	11.8
**Etnia/Color de piel**										
Blanca	55	27.0	39	22.0	0.266^‡^	76	25.7	18	21.2	0.396^‡^
No blanca	149	73.0	138	78.0		220	74.3	67	78.8
**Situación económica**										
Desempleado	36	17.7	57	32.2	0.001^‡^	71	24.0	22	25.9	0.720^‡^
Empleado	168	82.4	120	67.8		225	76.0	63	74.1
**Ingresos familiares (hasta 1 SM)^||^**										
>1 salario mínimo	169	82.8	129	72.9	0.019^‡^	241	81.4	57	67.1	0.005^‡^
≤1 salario mínimo	35	17.2	48	27.1		55	18.6	28	32.9
**Antecedentes familiares**										
No	110	53.9	68	38.6	0.003^‡^	149	50.5	29	34.1	0.008^‡^
Sí	94	46.1	108	61.4		146	49.5	56	65.9
**Comorbidades**										
No	174	85.3	141	80.6	0.221^‡^	252	85.1	63	75.9	0.047^‡^
Sí	30	14.7	34	19.4		44	14.9	20	24.1
**Enfermedades respiratorias**										
No	154	75.5	114	64.8	0.022^‡^	213	72.2	55	64.7	0.182^‡^
Sí	50	24.5	62	35.2		82	27.8	30	35.3
**Internación en hospital**										
No	199	98.0	172	98.3	0.854^§^	287	97.6	84	100.0	0.356^§^
Sí	4	2.0	3	1.7		7	2.4	0	0.0
**Antecedentes de trastornos mentales**										
No	192	95.1	136	77.7	<0.001^‡^	265	90.4	63	75.0	<0.001^‡^
Sí	10	5.0	39	22.3		28	9.6	21	25.0
**Fallecimiento de familiar(es) a raíz de COVID-19**										
No	166	81.8	136	76.8	0.235^‡^	242	81.8	60	71.4	0.039^‡^
Sí	37	18.2	41	23.2		54	18.2	24	28.6
**Fallecimiento de familiar(es) relacionado con otras enfermedades**										
No	134	66.7	125	71.4	0.320^‡^	205	70.2	54	64.3	0.302^‡^
Sí	67	33.3	50	28.6		87	29.8	30	35.7
**Temor de volver a contraer COVID-19**										
No	130	64.4	62	35.0	<0.001^‡^	168	57.1	24	28.2	<0.001^‡^
Sí	72	35.6	115	65.0		126	42.9	61	71.8
**Trabajó durante la pandemia**										
No	55	27.2	53	30.1	0.536^‡^	82	28.0	26	30.6	0.640^‡^
Sí	147	72.8	123	69.9		211	72.0	59	69.4
**Asistencia del gobierno**										
No	184	92.0	140	81.9	0.003^‡^	260	89.7	64	79.0	0.011^‡^
Sí	16	8.0	31	18.1		30	10.3	17	21.0
**Es el sustento económico de la familia**										
No	149	73.0	125	70.6	0.600^‡^	218	73.7	56	65.9	0.160^‡^
Sí	55	27.0	52	29.4		78	26.4	29	34.1

*n = Cantidad absoluta; ^†^% = Frecuencia relativa; ^‡^Chi-cuadrado; ^§^Prueba exacta de Fisher; ^||^Salario mínimo vigente = R$ 1320,00, Brasil, 2023

Los factores asociados a la ansiedad que se hicieron evidentes en los modelos de regresión indicaron que el temor de volver a contraer COVID-19 estuvo asociado a mayores probabilidades de ansiedad (Posible/Probable) (OR=3,08; IC95%: 1,95-4,88; p<0,001), al igual que a antecedentes previos de trastornos mentales (OR=5,56; IC95%: 2,42-12,76; p<0,001). La situación económica de los participantes presentó una asociación inversa, con menores probabilidades de ansiedad entre pacientes con trabajo, caracterizando un efecto protector y con una reducción aproximada del 46% en las probabilidades de sufrir ansiedad en relación con las demás categorías (OR=0,54; IC95%: 0,31-0,93; p=0,027). También se observó mayor probabilidad de ansiedad ante la presencia de antecedentes familiares de enfermedades crónicas (OR=1,76; IC95%: 1,11-2,79; p=0,016) (Tabla 4).

En lo que se refiere a la depresión, se hizo evidente una asociación con el temor de contraer COVID-19 (Posible/Probable), con OR=3,24 (IC95%: 1,85-5,69; p<0,001). Además, los participantes con antecedentes de trastornos mentales (OR=2,80; IC95%: 1,40-5,58; p=0,003), ingresos familiares de hasta un salario mínimo (OR=2,18; IC95%: 1,21-3,93; p=0,009), antecedentes familiares de enfermedades crónicas (OR=1,81; IC95%: 1,04-3,13; p=0,035) y presencia de comorbilidades (OR=1,97; IC95%: 1,03-3,78; p=0,041) presentaron mayores probabilidades de depresión, tal como se indica en la Tabla 4.

**Tabla 4:** Modelos de regresión logística binaria correspondientes a los factores asociados a la ansiedad y a la depresión entre pacientes afectados por COVID-19. Petrolina, PE, Brasil, 2023

Variables	Ansiedad				Depresión			
	OR* ajustado	IC95%^†^		Valor p	OR* ajustado	IC95%^†^		Valor p
Temor de volver a contraer COVID-19								
Sí vs^‡^ No	3,08	1,95	4,88	<0,001	3,24	1,85	5,69	<0,001
Antecedentes de trastornos mentales								
Sí vs^‡^ No	5,56	2,42	12,76	<0,001	2,80	1,40	5,58	0,003
Situación económica								
Empleado	0,54	0,31	0,93	0,027				
Antecedentes familiares de enfermedades crónicas								
Sí vs^‡^ No	1,76	1,11	2,79	0,016	1,81	1,04	3,13	0,035
Ingresos familiares^§^ de hasta 1 salario mínimo								
≤1 salario mínimo vs^‡^ >1 salario mínimo	1,69	0,96	2,97	0,071	2,18	1,21	3,93	0,009
Tiempo desde diagnóstico de COVID-19 (meses)								
Aumento de 1 mês	0,98	0,95	1,01	0,133				
Comorbilidades								
Sí vs^‡^ No					1,97	1,03	3,78	0,041

*OR - *Odds Ratio*; ^†^IC95% - Intervalo de Confianza del 95%; ^‡^vs - *Versus*; Prueba Hosmer-Lemeshow de ajuste del modelo: Ansiedad: chi^²^(8)=2,12 y p=0,9773; Depresión: chi²(8)=13,92 y p=0,0839; ^§^Salario mínimo vigente = R$ 1320,00, Brasil, 2023

## Discusión

En este estudio se identificó elevada prevalencia de síntomas de ansiedad y depresión en personas evaluadas después de la infección por SARS-CoV-2, indicando persistencia de alteraciones en la salud mental durante el período post-agudo de COVID-19. Estos resultados estuvieron asociados a características sociodemográficas, clínicas y psicosociales, entre las que se destacaron pertenecer al sexo femenino, vulnerabilidad socioeconómica y temor persistente de reinfección.

El valor de prevalencia de la ansiedad observado fue superior al aumento global estimado del 25% durante el primer año de la pandemia de COVID-19^3^; también fue mayor que las estimaciones descritas para la población brasileña en general antes y durante la pandemia, que indican una prevalencia aproximada del 14,3% para la ansiedad y del 4% al 5% en el caso de la depresión^15^ ^-^ ^16^. Este hallazgo constituye un aspecto relevante del estudio, puesto que pone de manifiesto que las personas que atravesaban el período post COVID-19 seguían presentando mayor riesgo de distrés psicológico en comparación con la población general. En contraste con un estudio de cohorte realizado en Brasil con pacientes internados en el que se utilizó el mismo instrumento, los valores de prevalencia encontrados fueron diferentes^10^, lo que puede estar relacionado con diferencias contextuales y metodológicas. La muestra de este estudio estuvo compuesta predominantemente por casos leves controlados en el contexto domiciliario, lo que puede haber favorecido la persistencia de síntomas psicológicos menos intensos clínicamente, aunque duraderos.

Los resultados demostraron ser coherentes con evidencias longitudinales internacionales. Estudios realizados con sobrevivientes de COVID-19 controlados después del tratamiento hospitalario identificaron valores de prevalencia más elevados para la ansiedad (42%) y la depresión (31%)^17^; esto sugiere que la gravedad clínica inicial puede modular estos desenlaces. Por otro lado, estudios de cohorte con seguimiento prolongado demostraron que, aunque los síntomas de la depresión tendieron a reducirse con el tiempo, los niveles de ansiedad permanecieron estables; esto indica que la ansiedad presenta un curso más persistente de la depresión durante el período post-COVID-19^18^. En este contexto, evidencias recientes indicaron que la ansiedad y la depresión tienden a manifestarse de forma concomitante y que actúan como factores predictivos de la condición post COVID-19, reforzando la hipótesis de que el distrés psicológico forma parte del espectro clínico del COVID prolongado^5^.

Además de los elevados valores de prevalencia, en los modelos de regresión logística se evidenció que el temor a un nuevo contagio de COVID-19 y los antecedentes previos de trastornos mentales fueron los principales factores asociados tanto a la ansiedad como a la depresión, aumentado aproximadamente tres veces las probabilidades de estos desenlaces. Adicionalmente, ingresos familiares de hasta un salario mínimo, antecedentes familiares de enfermedades crónicas y presencia de comorbilidades se asociaron a una mayor probabilidad de sufrir depresión, mientras que tener empleo demostró ser un factor de protección contra la ansiedad.

Se observó mayor prevalencia de ansiedad y depresión entre las mujeres, un resultado similar al descrito en estudios nacionales^10^ ^,^ ^17^ e internacionales^19^. Esta mayor vulnerabilidad en el sexo femenino a padecer trastornos mentales en comparación con los hombres también se identificó en la población brasileña durante la pandemia de COVID-19^17^. Este hallazgo se condice con informes globales que señalan un riesgo entre 1,5 y 2 veces mayor de padecer trastornos mentales entre las mujeres^3^. Además, en un metaanálisis reciente realizado con sobrevivientes de COVID-19 se identificaron más probabilidades de ansiedad (OR=2,1) y depresión (OR=1,8) entre las mujeres, lo que refuerza el hecho de que las desigualdades de género persistieron en el contexto pospandémico^19^. La mayor vulnerabilidad entre las mujeres se atribuyó a sobrecarga de actividades domésticas y de cuidado, a inseguridad económica y al impacto psicosocial extendido durante la pandemia^19^.

En lo que se refiere a los determinantes socioeconómicos, los hallazgos evidenciaron que tener empleo ejerció un efecto protector contra la ansiedad, mientras que ingresos familiares bajos aumentaron significativamente las probabilidades de sufrir depresión. Estos resultados se condijeron con los de estudios longitudinales recientes que demostraron que el desempleo elevó hasta 2,4 veces la probabilidad de ansiedad y que los bajos ingresos duplicaron el riesgo de depresión en seguimientos prolongados después de COVID-19^20^. De manera similar, en un estudio sobre evoluciones pospandémicas se identificó que ingresos familiares ≤1 salario mínimo aumentaron 2,1 veces la probabilidad de depresión, mientras que tener empleo mantuvo su efecto protector en torno al 40%, corroborando el patrón observado en este trabajo de investigación^21^.

Los antecedentes previos de trastornos mentales se destacaron como uno de los factores más profundamente asociados a la ansiedad y a la depresión; este resultado se condice con los de análisis longitudinales de tres años que indicaron elevada incidencia o persistencia de trastornos mentales después de COVID-19 en individuos con vulnerabilidad psiquiátrica previa^22^. Otras evidencias indicaron que personas con trastornos mentales previos presentaron un nivel de riesgo hasta cinco veces más alto de desarrollar síntomas de ansiedad y depresión en el contexto del COVID prolongado^23^.

De manera similar, la asociación entre enfermedades crónicas, comorbilidades y síntomas de depresión que se observó en este estudio se condijo con diversos metaanálisis que indicaron mayor probabilidad de ansiedad y depresión entre personas con enfermedades crónicas (con valores de OR de 1,7 a 2,0), posiblemente mediada por mecanismos de neuroinflamación persistente^5^. Otros estudios también demostraron que la presencia de comorbilidades prácticamente duplicó la probabilidad de depresión (OR=1,9) y que las enfermedades respiratorias aumentaron el riesgo de ansiedad (OR=1,6); esto refuerza la interacción entre afecciones físicas y distrés psicológico durante el período post COVID-19^24^.

En los modelos múltiples se demostró que los pacientes con antecedentes familiares de comorbilidades y personales de trastornos mentales presentaron mayores probabilidades de sufrir ansiedad y depresión, en consonancia con estudios epidemiológicos que señalan a los antecedentes psiquiátricos como un importante factor de vulnerabilidad para el desarrollo o la intensificación de trastornos psicológicos después de la infección^5^ ^,^ ^22^ ^-^ ^23^. Adicionalmente, la presencia de comorbilidades clínicas prácticamente cuadruplicó la probabilidad de depresión, reforzando el rol de las afecciones crónicas en la sobrecarga emocional de estos pacientes.

Además, el temor de volver a infectarse de SARS-CoV-2 presentó una fuerte asociación tanto con la ansiedad como con la depresión en este estudio. Un trabajo de investigación realizado en Mato Grosso do Sul verificó que el 64% de los participantes declararon tener temor de volver a contraer la enfermedad^24^, una condición que tiende a aumentar la carga emocional y física, favoreciendo el distrés psicológico. Estudios internacionales evidenciaron que el temor de volver a contraer COVID-19 surgió como el principal factor de predicción para la ansiedad y la depresión; este hallazgo se condice con trabajos de investigación sobre evoluciones de salud mental en los que se identificó un aumento de 2,8 a 3,5 veces en las probabilidades para ambos desenlaces asociados al temor persistente^21^. Un metaanálisis específico sobre factores psicológicos del COVID prolongado reforzó este resultado al demostrar que el temor a la reinfección aumentó aproximadamente tres veces la probabilidad de ansiedad, erigiéndose como uno de los determinantes más robustos del distrés psicológico prolongado^5^.

Las principales limitaciones de este estudio fueron las siguientes: su diseño transversal, que imposibilitó realizar inferencias causales; y el predominio de casos leves, que limitó la generalización de los resultados a poblaciones que presentan mayor gravedad clínica. Además, la escasez de estudios nacionales con un diseño similar restringió la posibilidad de establecer comparaciones más amplias.

A pesar de estas limitaciones, el estudio contribuyó a profundizar el conocimiento científico en las áreas de Salud y Enfermería porque produjo evidencias nacionales sobre la prevalencia y los factores asociados a la ansiedad y la depresión durante el período post COVID-19, en una población predominantemente conformada por casos leves controlados en un contexto no hospitalario. Al integrar variables sociodemográficas, clínicas y psicosociales en modelos analíticos ajustados, los hallazgos ampliaron lo que se conoce sobre los determinantes del distrés psicológico después de la infección por SARS-CoV-2. Estas evidencias fortalecen las bases científicas para el desarrollo de estrategias de detección, seguimiento y atención integral en salud mental, con repercusiones directas para la práctica asistencial, la planificación en salud y la producción de conocimientos de Enfermería.

## Conclusión

En este estudio se identificó elevada prevalencia de ansiedad entre sobrevivientes de COVID-19, con mayor probabilidad entre personas que tienen temor de volver a contagiarse, antecedentes personales de trastornos mentales, antecedentes familiares de enfermedades crónicas, bajos ingresos y situación de desempleo, mientras que tener trabajo demostró ejercer un efecto protector. La depresión presentó un valor de prevalencia menor al observado en el caso de la ansiedad, pero estuvo asociada principalmente al temor de reinfección, a los antecedentes de trastornos mentales, a los bajos ingresos, a los antecedentes familiares de enfermedades crónicas y a la presencia de comorbilidades clínicas.

Estos hallazgos refuerzan la necesidad de implementar estrategias de detección sistemática de síntomas de ansiedad y depresión en la población post COVID-19, enfocándose prioritariamente en grupos socialmente vulnerables y en personas con antecedentes psiquiátricos. También destacan la importancia de ofrecer intervenciones psicosociales que puedan reducir el impacto de COVID-19 sobre la salud mental.

## Data Availability

Todos los datos generados o analizados durante este estudio están incluidos en este artículo publicado.
